# Clinical significance of midkine expression in pancreatic head carcinoma

**DOI:** 10.1038/sj.bjc.6603879

**Published:** 2007-07-10

**Authors:** S Maeda, H Shinchi, H Kurahara, Y Mataki, H Noma, K Maemura, K Aridome, T Yokomine, S Natsugoe, T Aikou, S Takao

**Affiliations:** 1 Department of Surgical Oncology and Digestive Surgery, Kagoshima University, Graduate School of Medical and Dental Science, 8-35-1 Sakuragaoka, Kagoshima 890-8520, Japan; 2 Frontier Science Research Center, Kagoshima University Faculty of Medicine, 8-35-1 Sakuragaoka, Kagoshima 890-8520, Japan

**Keywords:** midkine, pancreatic carcinoma, immunohistochemistry, microvessel density, predicting factor

## Abstract

Midkine (MK) is a heparin-binding growth factor and a product of a retinoic acid-responsive gene. Midkine is overexpressed in many carcinomas and thought to play an important role in carcinogenesis. However, no studies have been focussed on the role of MK in pancreatic carcinoma. This study sought to evaluate the clinical significance of MK expression in pancreatic head carcinoma, including the relationship between immunohistochemical expression and clinicopathologic factors such as prognosis. Immunohistochemical expression of MK and CD34 was evaluated in pancreatic head carcinoma specimens from 75 patients who underwent surgical resection. Midkine was expressed in 53.3% of patients. Midkine expression was significantly correlated with venous invasion, microvessel density, and liver metastasis (*P*=0.0063, 0.0025, and 0.0153, respectively). The 5-year survival rate was significantly lower for patients positive for MK *vs* patients negative for MK (*P*=0.0073). Multivariate analysis revealed that MK expression was an independent prognostic factor (*P*=0.0033). This is the first report of an association between MK expression and pancreatic head carcinoma. Midkine may play an important role in the progression of pancreatic head carcinoma, and evaluation of MK expression is useful for predicting malignant properties of pancreatic head carcinoma.

Patients with adenocarcinoma of the pancreas have worse survival than patients with any other gastrointestinal malignancy (Silverberg and Lubera, 1988). The poor prognosis is principally due to difficulty in diagnosing pancreatic adenocarcinoma at a localised resectable stage and the propensity towards early tumour metastasis to regional lymph nodes and the liver. An important prognostic factor for pancreatic head carcinoma is the presence or absence of lymphatic and venous invasion. Without the ability to recruit new vessels, most tumours' growth is limited. Tumour angiogenesis is significantly correlated with tumour progression, invasion, and metastasis and recognised as an important contributor to poor prognosis (Weidner *et al*, 1991).

Midkine (MK) is a secreted heparin-binding growth factor with a molecular weight of 13 kDa. It is a product of a retinoic acid-responsive gene. Midkine and pleiotrophin (PTN) compose the heparin-binding growth-associated molecule family, which is distinct from other heparin-binding growth factor families (Kadomatsu *et al*, 1988; Tomomura *et al*, 1990). Midkine and PTN also lack similarity to other growth factors or cytokines (Salama *et al*, 2006). Midkine has the apparent ability to promote vascularisation and fibroblast growth, suppress apoptosis, and induce cell migration, and is thought to be involved in carcinogenesis and tumour progression (Choudhuri *et al*, 1997; Kadomatsu and Muramatsu, 2004). All of these studies suggest that MK plays an important role in carcinogenesis and the development and metastasis of tumours, and that it could serve as a novel tumour marker.

As MK is a secretory protein, its level in the blood can be monitored. In 87% of human adult carcinomas, serum MK is elevated; the level decreases after tumour removal (Ikematsu *et al*, 2000). In oesophageal carcinoma, urinary MK is elevated (Ikematsu *et al*, 2003), and high level of serum MK is associated with tumour progression (Obata *et al*, 2005), tumour size, rate of positivity for MK, MK immunoreactivity, and poor survival (Shimada *et al*, 2003).

MK expression is restricted to some tissues in normal adults. For example, expression of MK is high in the small intestine, moderate in the thyroid, and weak in the lung, colon, stomach, kidney, and spleen. However, none of MK expression is seen in normal liver. In the last few years, MK was found to be overexpressed in various human malignant tumours. Northern blot experiments revealed MK mRNA expression in gastric cancers (Aridome *et al*, 1995, 1998), colorectal cancers (Aridome *et al*, 1995, 1998; Ye *et al*, 1999), urinary bladder cancer (O'Brien *et al*, 1996), neuroblastoma (Nakagawara *et al*, 1995), Wilm's tumour (Tsutsui *et al*, 1993), and astrocytoma (Mishima *et al*, 1997). Interestingly, MK mRNA was reported to be extensively expressed in the early stage of colon cancer carcinogenesis (Ye *et al*, 1999). In gastric carcinoma, elevated expression of MK mRNA is significantly more prominent in well-differentiated and moderately differentiated adenocarcinoma than in poorly differentiated adenocarcinoma (Aridome *et al*, 1995). Furthermore, overexpression of MK mRNA is positively correlated with advanced tumours and poor prognosis, especially in the case of patients with neuroblastoma and bladder carcinoma (Nakagawara *et al*, 1995; O'Brien *et al*, 1996). Immunohistochemical studies revealed MK protein expression in other carcinomas, including oral carcinoma (Ruan *et al*, 2007), oesophageal carcinoma (Ren and Zhang, 2006), gastrointestinal stromal tumours (Kaifi *et al*, 2007), liver carcinoma (Kato *et al*, 2000b), lung carcinoma (Sakitani *et al*, 1999), thyroid carcinoma (Kato *et al*, 2000a), and prostate carcinoma (Konishi *et al*, 1999).

As mentioned above, MK is expressed in a variety of human malignant tumours, but to our knowledge no study has been focussed on correlation between MK expression and pancreatic head carcinoma, one of the most aggressive gastrointestinal carcinomas. The purpose of this study was (i) to examine the expression of MK in 75 cases of pancreatic head carcinoma by immunohistochemical methods, (ii) to explore possible correlation between MK expression and clinicopathologic variables, and (iii) to determine the prognostic value of MK expression.

## MATERIALS AND METHODS

In accordance with the institutional guidelines of our hospital, tissue specimens were collected after delivery of informed consent (Kurahara *et al*, 2004).

### Patients for histological and biochemical analyses

Formalin-fixed, paraffin-embedded blocks were obtained from 75 patients (50 male and 25 female patients) with invasive ductal adenocarcinoma of the pancreatic head carcinoma, who had received surgical treatment at Kagoshima University Hospital. All of the patients underwent macroscopically curative resection by pancreaticoduodenectomy (PD) with lymph node dissection. Patients had not received any preoperative chemotherapy or radiotherapy. Patient age ranged from 42 to 80 years (median 66.2 years). The number of patients with pT1, pT2, pT3, and pT4 tumours was 3 (4.0%), 5 (6.7%), 59 (78.7%), and 8 (10.7%), respectively.

All of the resected primary tumours and lymph nodes were histologically examined by haematoxylin and eosin staining using the tumour-node-metastasis classification system (Sobin, 2003). Histologically, all of the tumours were invasive ductal adenocarcinomas (29 well differentiated, 43 moderately differentiated, and 3 poorly differentiated). Lymphatic invasion was found in 66 tumours (88.0%) and venous invasion in 56 tumours (74.7%). Lymph node metastasis was found in 47 tumours (62.7%).

After discharge, all patients were followed up every 3 months with radiography, ultrasonography, and computed tomography. Usually, most recurrent liver disease was detected by computed tomography. New lesions detected by imaging were considered indicative of relapse. The median follow-up period was 20 months (ranging from 3 to 168 months). During these periods, 28 (37.3%) patients experienced recurrence of liver disease.

For Western blot analysis, primary pancreatic tumours and non-cancerous tissues were obtained by PD surgeries from the same patient. Namely, non-cancerous tissue was dissected from a region approximately 3 cm apart from the cancerous region.

### Cell culture

Four pancreatic carcinoma cell lines (PANC-1, MIA PaCa-2, Capan-1, and AsPC-1) were used. They were all provided by the American Type Culture Collection (Manassas, VA, USA). PANC-1, MIA PaCa-2, Capan-1, and AsPC-1 were derived from pancreatic tubular adenocarcinoma, pancreatic carcinoma, pancreatic adenocarcinoma after liver metastasis, and ascites of pancreatic adenocarcinoma, respectively. These cells were cultured in Dulbecco's modified Eagle's medium (Sigma-Aldrich Co. Ltd, St Louis, MO, USA) containing 10% fetal bovine serum (Sigma-Aldrich Co. Ltd) and 100 U ml^−1^ penicillin and streptomycin in a atmosphere of 5% CO_2_ in air.

### Immunohistochemical staining

Primary lesions were fixed in 10% formaldehyde, routinely embedded in paraffin, and cut into 3-*μ*m-thick sections. Sections were deparaffinised in xylene, rehydrated in graded series of ethanol, and incubated in 3.0% hydrogen peroxide (H_2_O_2_) in methanol for 10 min to block the endogenous peroxidases. The slides were autoclaved at 120°C for 10 min in 10 mM sodium citrate (pH 6.0) and cooled to room temperature. To block nonspecific reactions, sections were first incubated in normal rabbit serum (200-fold diluted; for staining by MK antibody) or normal horse serum (200-fold diluted; for staining by CD34 antibody) for 30 min at room temperature. They were then incubated overnight at 4°C with anti-MK antibody (human MK goat polyclonal antibody; Santa Cruz Laboratory, Santa Cruz, CA, USA) diluted 1 : 200 in phosphate-buffered saline (PBS) and anti-CD34 antibody (human CD34 mouse polyclonal antibody; Dako Corporation, Carpinteria, CA, USA) diluted 1 : 100 in PBS. The reactions were developed using the avidin–biotin immunoperoxidase technique (ABC method) (Hsu *et al*, 1981). Immunoreactivity was visualised using the Vectastain Elite ABC kit and a 3,3′-diaminobenzidine solution (Vector Laboratories Inc., Burlingame, CA, USA). Sections were then lightly counterstained with haematoxylin. For the positive control, sections known to be positive for MK were stained under the same conditions. For the negative control, sections were processed as mentioned above, except that the primary antibody was replaced by normal goat serum (200-fold diluted). Anti-MK immunoreactivity was confined primarily to the cytoplasm. All immunostained slides were evaluated by two independent observers (SM and ST). Ten fields were randomly selected, and expression in 1000 tumour cells (100 cells per field) was evaluated with high-power (× 200) microscopy. First, we classified the specimens stained by anti-MK antibody with a visual grading system employed for immunohistochemical evaluation (Moon *et al*, 2003). Samples were considered negative if less than 10% of the cells are stained for MK; weak was defined as 10–25% of the tumour staining positive; moderate as 25–50% staining, and strong as more than 50% of the tumour staining positive. More than 10% positive staining was defined as positive expression.

### Microvessel counting

We used in this study anti-CD34 antibody for counting microvessels, because many articles published had employed CD34 immunostaining for analysing microvessel densities (MVD). Vessels in the five most highly vascularised areas (0.785 mm^2^ per field) visualised by CD34 immunostaining were counted under a light microscope (with × 200 power (× 20 objective and × 10 ocular)). The MVD was determined using a method previously described (Weidner *et al*, 1991). The tumour MVD was calculated as the mean value for five fields. Tumours with MVD⩾40 or <40 were classified as having high-grade and low-grade vascularity, respectively (Figure 3).

### Western blotting

Whole-cell lysates were prepared according to the Santa Cruz protocol. Glysates (12 *μ*g) were subjected to immunoblot analysis using a 15% SDS–polyacrylamide gel and electrotransferred onto nitrocellulose filters (Bio-Rad Laboratories, Hercules, CA, USA). The filters were incubated with MK antibody (diluted to 1 : 200 in PBS) followed by peroxidase-conjugated anti-goat IgG antibody (diluted to 1 : 2000 in PBS; Santa Cruz Laboratory) as a secondary reaction. As an internal control for the amount of protein loaded, a portion of filters was reacted with anti-*β*-actin antibody (diluted to 1 : 200 in PBS; Santa Cruz Laboratory). After incubation, they were then reacted with peroxidase-conjugated anti-goat IgG antibody, as mentioned above. The immunocomplex was visualised using the ECL Western blot detection system (Pierce, Rockford, IL, USA). At least three independent experiments were performed.

### Statistical analyses

Statistical analysis of group differences was performed using the *χ*^2^ test. The Kaplan–Meier method was used to analyse survival after surgical treatment of patients, and the log-rank test was used to estimate differences in survival. Prognostic factors were examined using univariate and multivariate analyses (Cox proportional hazards regression model). *P*<0.05 was considered statistically significant. All statistical analyses were performed using StatView statistical software version 5.0 (SAS Institute Inc., Cary, NC, USA).

## RESULTS

### Expression of MK in pancreatic head carcinoma

**Figure 1:**
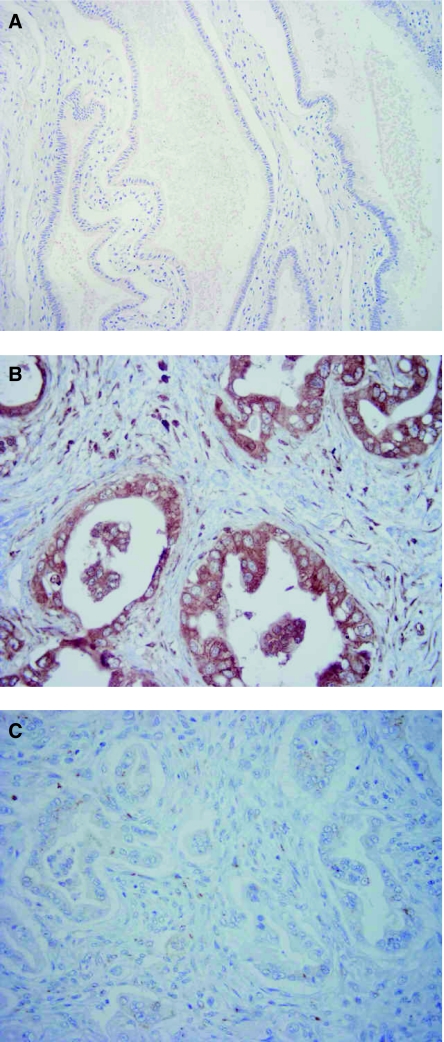
Immunohistochemical staining for MK in invasive ductal adenocarcinoma of the pancreas head. (**A**) Normal pancreatic ductal epithelium dissected approximately 3 cm apart from the cancerous region (× 200). Note that almost all cells are unstained or stained very slightly by the antibody. (**B**) Carcinoma cells positively stained by MK antibody (× 400). Note cytoplasmic staining for MK. (**C**) Carcinoma cells stained negatively by MK antibody (× 400).

In 40 of 75 patients (53.3%), positive expression of MK was observed in the cytoplasm of carcinoma cells (Figure 1B). Notably, the intensity of MK expression was stronger in areas abundant in vessels and in the invading border of tumours. However, negative expression of MK was also seen in some (46.7%, 35 out of 75) of pancreatic head carcinomas examined (Figure 1C). No immunoreactivity or only a slight staining for MK was noted in normal pancreatic ductal epithelium, which was approximately 3 cm apart from the cancerous region (Figure 1A).

### Western blot analysis of MK expression

**Figure 2:**
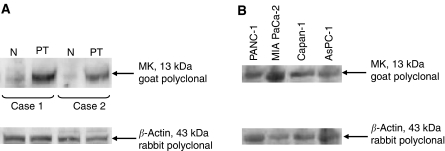
Western blotting for MK protein. (**A**) Pancreatic primary tumours (PT) and non-cancerous regions (N) dissected approximately 3 cm apart from the cancerous region. Cases 1 and 2 are from each independent patient. (**B**) Pancreatic carcinoma cell lines. Note the presence of a 13-kDa band corresponding to MK protein in primary tumours and cultured cells. The presence of a 43-kDa band corresponding to *β*-actin protein in each sample indicates that samples are equally loaded.

Western blot analysis demonstrated that MK protein was strongly expressed in the primary pancreatic carcinomas obtained from two independent patients. In contrast, in the non-cancerous pancreatic tissues of the same patients only a little amount of MK protein was synthesised (Figure 2A). Strong expression of MK protein was also seen in all four pancreatic carcinoma cell lines (PANC-1, MIA PaCa-2, Capan-1, and AsPC-1) tested (Figure 2B).

### Correlation between MK expression and clinicopathologic factors

MK expression was significantly associated with the clinicopathologic parameters, including venous invasion, liver metastasis, and MVD grade. In the group with MK-positive tumours, there was a significantly higher incidence of venous invasion (*P*=0.0063), liver metastasis (*P*=0.0153), and MVD grade (*P*=0.0021) (Table 1). However, there was no significant association between expression of MK and age, gender, histologic type, tumour depth, cancer stage, lymphatic invasion, or lymph node metastasis (Table 1).

### Relationship between MVD and MK expression

**Figure 3:**
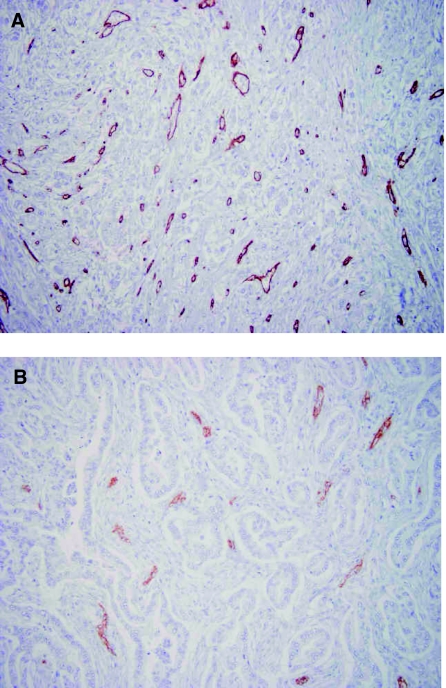
Immunohistochemical staining for CD34 protein in invasive ductal adenocarcinoma of the pancreas head. (**A**) A microphotograph of pancreatic head carcinoma stained by CD34 antibody and judged as high-grade MVD⩾40 (× 200). (**B**) A microphotograph of pancreatic head carcinoma stained by CD34 antibody and judged as low-grade MVD<40 (× 200).

**Figure 4:**
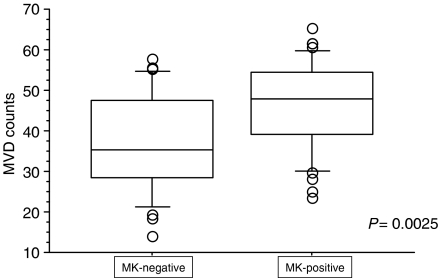
Microvessel density (MVD) evaluated by CD34 staining and subsequent observation (0.785 mm^2^ per field) under a microscope with × 200 power. The MVD (mean±s.d.=45.72±10.77) in MK-positive tumours was significantly higher than that (36.69±11.91) in MK-negative tumours (*P*=0.0025).

Microvessels are heterogeneously distributed within tumours, and the MVD is generally greatest at the invading border of tumours. When MVD was evaluated by staining specimens with CD34 antibody, the number of CD34-positive blood endothelial cells varied among tumour specimens. In Figure 3A, a tumour with high-grade MVD is shown as a typical sample. On the other hand, a tumour with low-grade MVD is shown in Figure 3B. The median MVD was 41.50±11.21 (ranging from 14.1 to 65.3 per field). Notably, the MVD was significantly (*P*=0.0025) higher in the MK-positive tumours (mean±s.d.=45.72±10.77) than in the MK-negative tumours (36.69±11.91; Figure 4).

### Prognostic impact of MK expression

**Figure 5:**
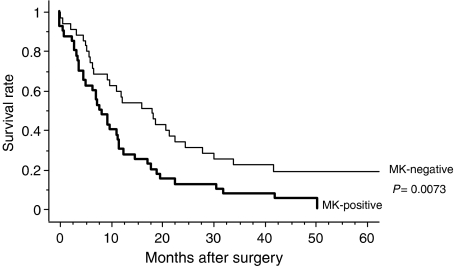
Comparison of survival curves (depicted according to the Kaplan–Meier method) of patients with pancreatic head carcinomas in relation to MK expression. The 5-year survival rate was 0.0% for patients with MK-positive tumours and 19.3% for patients with MK-negative tumours, which is significantly different (*P*=0.0073).

The 5-year survival rate of patients with tumours positive for MK expression was 0.0%, whereas the rate for patients with tumours negative for MK expression was 19.3%. There was a significant difference in the 5-year survival rate between patients with tumours positive and negative for MK expression (*P*=0.0073; Figure 5).

### Univariate and multivariate survival analyses

Tables 2 and 3 show the results of univariate and multivariate analyses of factors related to patient prognosis. Univariate analysis demonstrated that the factors including lymphatic invasion, lymph node metastasis, tumour depth, cancer stage, and MK expression were significantly (*P*<0.05) related to postoperative survival. Multivariate regression demonstrated that lymph node metastasis and MK expression were independent prognostic factors, while lymphatic invasion and tumour depth were not.

## DISCUSSION

Many studies have shown that growth factors not only promote tissue proliferation, but also induce malignant transformation. In another word, they involve in the development of neoplasm. In fact, various growth factors are reported to be overexpressed in many human tumours. Like other growth factors, MK is known to promote cell survival (Qi *et al*, 2000), cell growth (Takei *et al*, 2001, 2005), and cell migration (Sato *et al*, 2001). These biological activities support the hypothesis that MK may involve in oncogenesis and tumour progression.

Although the biological role of MK in tumour growth and progression is not fully understood, MK is thought to be a novel molecular mediator of tumour angiogenesis (Choudhuri *et al*, 1997; Muramatsu, 2002). This angiogenic role of MK in tumourigenesis evokes us to suppose that MK expression is also involved in growth and progression of a tumour (Kadomatsu and Muramatsu, 2004). To explore the possible correlation between MK expression and tumour progression, we examined MK expression using pancreatic head carcinomas and its cell lines. Western blot experiments revealed that MK was in fact strongly expressed in four pancreatic carcinoma cell lines (PANC-1, MIA PaCa-2, Capan-1, and AsPC-1) tested. Midkine expression was also seen in surgically resected pancreatic specimens. However, normal pancreatic tissue expressed only a slight amount of MK (Figure 2A). These findings suggest a possible involvement of MK in pathogenesis and progression of pancreatic head carcinoma, as previously suggested for other carcinomas (Aridome *et al*, 1995; O'Brien *et al*, 1996; Moon *et al*, 2003).

Using 75 pancreatic head carcinomas, we investigated immunohistochemically whether MK expression was associated with clinicopathologic factors including prognosis. Midkine expression was observed in 53.3% of tumours. This rate was consistent with previous immunohistochemical data obtained from other carcinomas, where MK expression had been detected in 32.0–86.3% of tumours (Konishi *et al*, 1999; Kato *et al*, 2000a, 2000b; Ren and Zhang, 2006; Kaifi *et al*, 2007; Ruan *et al*, 2007). In this study, MK expression was found to be significantly associated with venous invasion, MVD, and liver metastasis. These features appear to be slightly different from those of other carcinomas. For example, in oral squamous cell carcinoma, the expression of MK was significantly correlated with tumour size, clinical stage, MVD, and vascular endothelial growth factor (VEGF) expression (Ruan *et al*, 2007). In oesophageal carcinoma, MK is more intensely expressed in well-differentiated tumours than in poorly differentiated tumours (Ren and Zhang, 2006). In hepatocellular carcinoma, no significant differences in MK expression were found among tumours with different histologic types (Kato *et al*, 2000b).

High-grade MVD was more often found in MK-positive tumours, reflecting a possible effect of MK on tumour vascularity in pancreatic head carcinoma. Consistent with these results, transfection of the breast carcinoma line MCF-7 with MK accelerates tumour growth and increases tumour vascularity after implantation of MK-overexpressing MCF-7 cells into nude mice (Choudhuri *et al*, 1997). Another angiogenic factors including VEGF, basic fibroblast growth factor, and platelet-derived endothelial cell growth factor appear to be of interest to test whether they are correlated with MK-positive tumours, although we have not yet tested these possibilities.

In this study, univariate analysis identified lymphatic invasion, lymph node metastasis, tumour depth, cancer stage, and MK expression as prognostic factors. In addition, multivariate analysis revealed that lymph node metastasis and MK expression were determined as independent prognostic factors. It was recently demonstrated that the expression of MK is associated with poor survival in oral squamous cell carcinoma (Ruan *et al*, 2007). In gastrointestinal stromal tumours, MK overexpression is an independent prognostic factor associated with poor prognosis (Kaifi *et al*, 2007). In oesophageal carcinoma, high level of serum MK is associated with tumour size, positivity rate, MK immunoreactivity, and poor survival and defined as an independent prognostic factor (Shimada *et al*, 2003).

In conclusion, MK expression in pancreatic head carcinoma was associated with venous invasion, liver metastasis, MVD, and prognosis, and defined as an independent prognostic factor. This is the first report of an association between MK expression and pancreatic head carcinoma. In this context, MK may be useful as a new diagnostic and prognostic biomarker in predicting malignant properties of pancreatic head carcinoma. Furthermore, suppression of MK gene expression using a recently developed RNAi technology during pathogenesis and progression of pancreatic head carcinoma may be an interesting trial for possible inhibition of this disease.

## Figures and Tables

**Table 1:** Correlation between MK expression and clinicopathologic factors in pancreatic head carcinoma

		**MK expression**		
	**Total *n*=75**	**Positive *n*=40 (53.3%)**	**Negative *n*=35 (46.7%)**	***P*-values**
*Age (years)*				
Mean±s.d.		67.4±8.9	64.9±9.9	0.2648
				
*Gender*				
Male	50 (66.7)	23 (57.5)	27 (77.1)	0.0718
Female	25 (33.3)	17 (42.5)	8 (22.9)	
				
*Histology*				
Well	29 (38.7)	14 (35.0)	15 (42.9)	0.2318
Moderately	43 (57.3)	23 (57.5)	20 (57.1)	
Poor	3 (4.0)	3 (7.5)	0 (0.0)	
				
*pT*				
pT1	3 (4.0)	1 (2.5)	2 (5.7)	0.3699
pT2	5 (6.7)	1 (2.5)	4 (11.4)	
pT3	59 (78.7)	33 (82.5)	26 (74.3)	
pT4	8 (10.7)	5 (12.5)	3 (8.6)	
				
*pN*				
Negative	28 (37.3)	14 (35.0)	14 (40.0)	0.6552
Positive	47 (62.7)	26 (65.0)	21 (60.0)	
				
*Liver metastasis*				
Negative	47 (62.7)	20 (50.0)	27 (77.1)	0.0153
Positive	28 (37.3)	20 (50.0)	8 (22.9)	
				
*pStage*				
I	6 (8.0)	1 (2.5)	5 (14.3)	0.3651
IIA	21 (28.0)	13 (32.5)	8 (22.9)	
IIB	38 (50.7)	20 (50.0)	18 (51.4)	
III	6 (8.0)	4 (10.0)	2 (5.7)	
IV	4 (5.3)	2 (5.0)	2 (5.7)	
				
*Lymphatic invasion*				
Negative	9 (12.0)	4 (10.0)	5 (14.3)	0.5688
Positive	66 (88.0)	36 (90.0)	30 (85.7)	
				
*Venous invasion*				
Negative	19 (25.3)	5 (12.5)	14 (40.0)	0.0063
Positive	56 (74.7)	35 (87.5)	21 (60.0)	
				
*Microvessel density*				
Low grade	33 (44.0)	11 (27.5)	22 (62.9)	0.0021
High grade	42 (56.0)	29 (72.5)	13 (37.1)	

MK=midkine; s.d.=standard deviation.

**Table 2:** Univariate analysis of prognostic factors in pancreatic head carcinoma

**Variables**	***n***	**5-year survival rate (%)**	***P*-values**
*Age (years)*			
Over 65	45	10.0	0.3578
Under 64	30	8.9	
			
*Gender*			
Male	50	10.0	0.8857
Female	25	0.0	
			
*pT*			
pT1, 2	8	37.5	0.0470
pT3, 4	67	5.5	
			
*pN*			
Negative	28	20.0	0.0036
Positive	47	2.8	
			
*Liver metastasis*			
Negative	47	13.1	0.1522
Positive	28	3.6	
			
*pStage*			
I, II	65	11.0	0.0136
III, IV	10	0.0	
			
*Lymphatic invasion*			
Negative	9	33.3	0.0297
Positive	66	6.4	
			
*Venous invasion*			
Negative	19	15.8	0.3625
Positive	56	6.7	
			
*Microvessel density*			
Low grade	33	10.6	0.1822
High grade	42	9.5	
			
*Midkine expression*			
Negative	35	19.3	0.0073
Positive	40	0.0	

**Table 3:** Multivariate analyses of prognostic factors in pancreatic head carcinoma

**Independent factors**	**Univariate *P*-values**	**Multivariate *P*-values**	**Hazard ratio**	**95% confidence interval**
*pT*				
pT1, 2/pT3, 4	0.0470	0.7197	1.198	0.446–3.220
				
*pN*				
Negative/positive	0.0036	0.0235	2.003	1.098–3.653
				
*Lymphatic invasion*				
Negative/positive	0.0297	0.4287	1.458	0.573–3.562
				
*Midkine expression*				
Negative/positive	0.0073	0.0033	2.143	1.290–3.711
